# Supplementary material for: Is transcutaneous auricular vagus nerve stimulation effective and safe for primary insomnia? A PRISMA-compliant protocol for a systematic review and meta-analysis
Source: PLoS One. 2024 Nov 25;19(11):e0313101. doi: 10.1371/journal.pone.0313101 (PMC11588257; doi:10.1371/journal.pone.0313101)
Supplement: S2 File — (PDF) [file pone.0313101.s002.pdf]

Supplementary file 2: Search strategies in other databases

Embase (via Ovid)

#1 'randomized controlled trial'/exp OR 'controlled clinical trial'/exp

#2 random*.ab,ti OR RCT.ab,ti

#3 #1 OR #2

#4 'human'/exp

#5 #3 AND #4

#6 'insomnia'/exp

#7 insomnia.ab,ti OR sleep disorder.ab,ti OR difficulty sleeping.ab,ti OR trouble sleeping.ab,ti

#8 #6 OR #7

#9 'vagus nerve stimulation'/exp

#10 #5 AND #8 AND #9

Cochrane Library

#1 (Randomized controlled trial OR Controlled clinical trial) [Publication Type]

#2 "Random* [Title/Abstract/Keywords]

#3 "RCT" [Title/Abstract/Keywords]

#4 #1 OR #2 OR #3

#5 (insomnia OR sleep disorder OR difficulty sleeping OR trouble sleeping) [Title/Abstract/Keywords]

#6 Vagus Nerve Stimulation [Title/Abstract/Keywords] OR VNS [Title/Abstract/Keywords] OR taVNS [Title/Abstract/Keywords] OR ta-VNS [Title/Abstract/Keywords]

#7 #4 AND #5 AND #6

PsycINFO

#1 Random* OR controlled [Abstract /Title/Keywords]

#2 RCT [Abstract /Title/Keywords]

#3 #1 OR #2

#4 insomnia OR sleep disorder OR difficulty sleeping OR trouble sleeping [Abstract /Title/Keywords]

#5 vagus nerve stimulation OR VNS OR taVNS OR ta-VNS OR iVNS [Abstract/Title/Keywords]

#6 #3 AND #4 AND #5

AMED

#1 Random*.mp. OR RCT.mp.

#2 insomnia.mp. OR sleep disorder.mp.

#3 Vagus Nerve Stimulation.mp. OR “VNS”.mp. OR “taVNS”.mp. OR “iVNS”.mp.

#4 #1 AND #2 AND #3

PEDro

#1 insomnia OR sleep disorder OR difficulty sleeping OR trouble sleeping[Abstract/Title]

#2 vagus nerve stimulation OR VNS OR taVNS OR ta-VNS OR iVNS [Abstract/Title]

#3 clinical trial [Method]

#4 #1 AND #2 AND #3

Wangfang Database

#1 随机 [标题/摘要]

#2 对照 [标题/摘要]

#3 #1 OR #2

#4 失眠 OR 睡眠障碍 OR 入睡困难 OR 睡眠紊乱 OR 不寐 [标题/摘要]

#5 迷走神经刺激 OR 经皮耳穴迷走神经刺激 OR 耳迷走神经刺激 OR 迷走神经 [标题/摘要]

#6 VNS OR taVNS OR ta-VNS [标题/摘要]

#7 #5 OR #6

#8 #3 AND #4 AND #7

Chinese National Knowledge Infrastructure (CNKI)

#1 随机 [标题/摘要]

#2 对照 [标题/摘要]

#3 #1 OR #2

#4 失眠 OR 睡眠障碍 OR 入睡困难 OR 睡眠紊乱 OR 不寐 [标题/摘要]

#5 迷走神经刺激 OR 经皮耳穴迷走神经刺激 OR 耳迷走神经刺激 OR 迷走神经 [标题/摘要]

#6 VNS [标题/摘要] OR taVNS [标题/摘要] OR ta-VNS [标题/摘要]

#7 #5 OR #6

#8 #3 AND #4 AND #7

Chinese BioMedical Literature Database (CBM)

#1 随机 [标题/摘要]

#2 对照 [标题/摘要]

#3 #1 OR #2

#4 失眠 OR 睡眠障碍 OR 入睡困难 OR 睡眠紊乱 OR 不寐 [标题/摘要]

#5 迷走神经刺激 OR 经皮耳穴迷走神经刺激 OR 耳迷走神经刺激 OR 迷走神经 [标题/摘要]

#6 VNS OR taVNS OR ta-VNS [标题/摘要]

#7 #5 OR #6

#8 #3 AND #4 AND #7
